# External validity in translational biomedicine: understanding the conditions enabling the cause to have an effect

**DOI:** 10.15252/emmm.202114334

**Published:** 2021-12-20

**Authors:** Ulrich Dirnagl, Alexandra Bannach‐Brown, Sarah McCann

**Affiliations:** ^1^ Department of Experimental Neurology Charité ‐ Universitätsmedizin Berlin Berlin Germany; ^2^ QUEST Center for Responsible Biomedical Research Berlin Institute of Health Berlin Germany

**Keywords:** History & Philosophy of Science, Methods & Resources

## Abstract

A spectre is haunting biomedical research: It appears that a substantial fraction of published research results cannot be reproduced, while spectacularly successful novel treatments developed in experimental models of disease too often fail in clinical trials. A reproducibility crisis has been proclaimed, and bench‐to‐bedside translation appears to be lost in a “valley of death”. Both predicaments, non‐reproducibility and translational roadblocks, are connected: Why should we expect to successfully “trans‐late” results to humans, if already “cis‐lation”—that is, the generalization from one experimental setting to an identical or fairly similar one—often fails?

## Graphical abstract

This Commentary discusses how the neglect of external validity may have contributed to the failures to independently reproduce biomedical research results and the high attrition rate of bench to bedside translation.

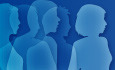

The best material model of a cat is another, or preferably the same, cat.

Norbert Wiener (1945)

During the past decade, a plethora of studies and commentaries have pinpointed a number of potential causes for the “reproducibility crisis” (Baker, 2016): various forms of uncontrolled biases, low sample sizes, questionable statistics, undisclosed freedom of researchers in experimental design, analysis and reporting, non‐publication of unwanted results, inappropriate study designs or patient selection in clinical trials, to name but a few (Macleod & Mohan, 2019). Overall, low internal validity emerges as a key culprit. Internal validity refers to the extent to which the results of a given study can be attributed to the effects of the experimental intervention, rather than some other, unrelated factors. Statistical conclusion validity, which is threatened by low statistical power, missing corrections for multiple testing and practices like p‐hacking or HARKing, among others, has also been in the spotlight. However, external validity, the extent to which the results of a given study hold when applied to other study conditions, (non‐human) animal strains/species, or humans, has received less attention. In the following, we will argue that this neglect of external validity may have substantially contributed to the failures to independently reproduce research results, and importantly, the exceedingly high attrition rate of bench‐to‐bedside translation (Gehr & Garner, 2016).

## External validity and the problem of induction

Apart from a simple lack of awareness—education and practice in biomedicine rarely involve epistemological reflection—an important reason for the current focus on low internal rather than low external validity may lie in the problem of induction. The truth of an inductive argument, which makes broad generalizations from specific observations, can only be tentative or probable, based upon the evidence given. Since the work of 18^th^‐century philosopher David Hume, induction is considered to have low inferential value. When we generalize from a model system, such as a rodent model of disease, to patients, we make inferences about a target system (humans) that we cannot study directly (Reiss, 2018). As a consequence, external validity is difficult to address and never completely answerable. Compare this to internal validity, which is the condition *sine qua non* of every meaningful experiment. Internal validity can be deduced or even measured in any given experimental setting and is, at least theoretically, fully under our control (Campbell, 1957). While the factors impacting internal validity are mostly known knowns, external validity is confounded by multiple known unknowns, as well as unknown unknowns. In the following, we will provide an overview of known threats to external validity, and end by trying to answer the question of how much external validity is necessary at certain stages of preclinical biomedical research—from pure basic to directly informing clinical trials.

## Multiple causes of low external validity

Probably to many, the most surprising and least understood threat to external validity is the standardization of environmental variables. Generally, variability of experimental results has biological, as well as random noise and measurement error components. Biological variation emerges from a combination of environmental factors and their interaction with genetic factors (“phenotypic plasticity”). Through environmental standardization, we try to minimize variability and increase internal validity. However, environmental factors are too numerous (from the perfume of those handling the animals to the composition of microbiota), and many of them remain obscured or are not under our control. Standardizing known environmental factors (e.g. temperature, humidity, time of the day, personnel) reduces the variation of experimental results in a given laboratory. Paradoxically, repeating the identical experiment in a different setting or laboratory may lead to statistically significant different results. Unknown environmental factors that differ between laboratories may affect the population mean—uncovering the fact that a true population mean is fiction and experimenters have, contrary to their intention and conviction, standardized to different environmental conditions. Würbel and colleagues have termed this the “standardization fallacy”: instead of making research results more robust and reproducible, environmental standardization often decreases external validity.

Better known and generally appreciated threats to external validity result from limiting experiments to animals of one sex or specific age groups. Sex and age, and their interaction, have a strong influence on (patho)physiology and pharmacology. Disease models can have different phenotypes in either sex, and experimental treatments that work in one sex may be ineffective in the other, just as in humans. Similarly, disease phenotypes, outcomes or treatments studied in the same model may differ markedly between young and old animals. Disturbingly, however, entire biomedical fields are still biased towards using either female or male individuals in their research. For example, while cardiovascular researchers preferentially use male animals, infection biology is studied more often in female animals (Flórez‐Vargas *et al*, 2016). In the absence of any biological reasoning for such sex biases, it is very likely that they must be explained historically: Today’s scientists still work with sex‐specific models developed by pioneers in the field, and often (erroneously) believe that female animals introduce extra variability into experimental results. Since ageing animals and maintaining them is costly, and aged animals become frail and thus disease phenotypes more severe, just as in patients, most experimental research on animals is biased towards the use of adolescents or young adults, even in fields that study diseases primarily affecting the elderly.

An additional threat to external validity particularly relevant to research on diseases prevalent in the elderly, such as stroke or dementia, is comorbidities. Often elderly patients suffer from several conditions simultaneously, such as hypertension, diabetes or obesity. Since in these cases multiple disease pathologies interact, modelling only the target disease of interest may generate results that are not generalizable to populations with multiple pathologies.

Tightly connected with ageing is the influence of the immune system on external validity. As in humans, the immune system of animals matures with antigen and pathogen encounters until it deteriorates into immunosenescence. However, laboratory animals are kept under abnormally hygienic conditions (e.g. specific pathogen free (SPF) husbandry), one of the most drastic forms of environmental standardization (see above). Only recently, researchers realized that this prevents the immune system of these animals from maturing and ageing. This became obvious when clean mice, which exhibit a neonatal‐like immune status, were compared to mice bought in pet shops or caught in the wild, both of which have mature immune systems similar to adult humans (Beura *et al*, 2016). The majority of rodent studies are conducted under clean conditions, meaning even in aged rodents, diseases are modelled in the context of a neonatal immune status. This may have dramatic consequences as researchers are finding that the immune system contributes to pathology in almost every disease. It is hard to believe that results obtained using SPF rodents to study adult diseases such as Alzheimer’s disease, diabetes or atherosclerosis are not confounded by immune phenotypes and therefore, cannot be generalized to the relevant human populations. We speculate that the low external validity afforded by SPF housing is at least partly responsible for the exceedingly high rate of failures when attempting to translate results from rodents to humans.

Only recently, the microbiome came under the spotlight as a major modulator of (patho)physiology. Given that microbiota engage in intense crosstalk with the immune system and exert a plethora of effects on bodily systems and functions, it is not surprising they represent another important determinant of external validity. Microbiota can influence immunophenotypes and explain differences in disease model outcomes between identical mouse strains obtained from different breeders (e.g. (Ivanov *et al*, 2008; Sadler *et al*, 2017)). Not only are microbiota idiosyncratic to specific commercial breeders, their composition is modulated by the interaction between animal husbandry factors such as diet, caging and bedding (Ericsson *et al*, 2018). Further, as with immune phenotypes, microbiota from laboratory mice vary from those found in the wild, exhibiting reduced complexity and thus translational value.

Laboratory animals are fed ad libitum on diets that are formulated to provide rapid growth, health and reproductive fitness. Unlike the diets of wild animals, laboratory diets are loaded with vitamins, minerals, amino acids, etc., and often contain unspecified levels of hormone‐like compounds called phytoestrogens. Such diets can modulate the onset of puberty, pathologies and the impact of drugs, toxins and experimental interventions, among other effects. Compare this to humans, who often indulge in rather unhealthy diets. Exercise, or the lack thereof, may be another complicating factor. Without a running wheel, rodents kept in standard laboratory cages are sedentary. Like the food ad libitum regime, this may be reflective of a substantial proportion of the human population. Conversely, rodents provided with a running wheel exercise, covering distances similar to wild animals. Since exercise affects numerous physiological functions, from cardiovascular to neuroneogenesis, the generalizability of experimentally obtained results in disease models must consider housing conditions, along with diets, relative to the lifestyle and socioeconomic status of the human target population.

Individual disease phenotypes and treatment outcomes, as well as the effects of all the factors threatening external validity discussed above, are modulated, if not controlled, by the genetic makeup of the animals used. Human populations are genetically highly diverse, while most rodent strains used in biomedical research are inbred. While far from being completely isogenic, they are genetically very homogenous. In fact, this is why researchers use inbreds: to provide a standardized genetic background on which the effect of specific genes or interventions can be isolated, providing another example for the tension between standardization and external validity.

## How to increase external validity

Researchers, especially when interested in exploring disease mechanisms relevant in humans and developing novel therapies, can partially overcome this tension by prioritizing external validity over standardization. Every factor threatening generalizability discussed above may be targeted to increase external validity and the potential for reproducibility and successful translation (Table 1). To mention just a few examples: Research can be conducted in aged animals, animals with comorbidities, (diversity) outbred rodents or those raised on atherogenic diets. The immune system and microbiota can be induced to phenocopy human immune responses by generating so‐called "wildlings", which have natural microbiota and pathogens at all body sites while maintaining the tractable genetics of standard inbred mice (Rosshart *et al*, 2019). Environmental conditions can be systematically heterogenized, for example, by multi‐laboratory designs (Richter *et al*, 2010).

**Table 1:** Measures to help overcome known threats to external validity.

Known threats to external validity	Measures to increase external validity
Standardization of environmental variables, e.g. animal housing, husbandry	Introduce heterogeneity by splitting experiments into multiple replicates, systematically varying factors within laboratories and carrying out experiments across different laboratories
Age and sex of experimental animals	Select animals of the appropriate age(s) and sex(es) with respect to the human population under consideration
Healthy status of experimental animals	Where the disease of interest occurs in comorbid human populations, also model these comorbidities in animals
Immature immune phenotypes in laboratory animals caused by abnormally hygienic conditions, e.g. SPF	Implement animal breeding, housing and husbandry protocols that result in more mature immune phenotypes
Microbiota composition of experimental animals	Implement animal breeding, housing and husbandry protocols that result in microbiota more similar to wild animals; use animals from different batches or breeders to introduce heterogeneity
Diet and lifestyle factors, e.g. level of exercise	Implement feeding and exercise regimens that mimic the human population under consideration
Standardized genetic background of experimental animals	Use outbred animals and/or animals from different batches or breeders

These measures to maximize external validity may come at the cost of reduced precision and internal validity. For some factors, like animal age and sex, the effects may be minor. For others, the balance in this trade‐off may shift depending on the research type. In basic research aimed at answering fundamental (patho)physiological questions, it may be more important to prioritize standardization and reduce confounding factors (e.g. genetic and microbiota diversity) to gain maximum knowledge of the mechanism under study. It should be noted that irreproducibility or non‐translation of results in these situations can provide important insights into the influence of factor variants on (patho)physiological mechanisms. In contrast, in translational research external validity is paramount and identifying the mechanisms behind a successful therapy is generally not the primary aim.

We have adopted a broad definition of external validity that relates to generalizing findings from animals to humans across domains. Many descriptors related to experimental validity are derived from psychological test theory and are currently used inconsistently in the preclinical space. While not the focus of this article, additional aspects of experimental validity important for generalization, variously termed construct, predictive and translational validity, among others, are also critical components of successful translation. These terms usually relate to how well an animal model mimics the human disease of interest, or how well measured variables map onto underlying constructs (preprint: Esterling *et al*, 2021). For example, while establishing an immune phenotype of similar maturity to humans will increase external validity, known and unknown species and strain differences in immune responses might still affect generalizability in certain contexts. Again, these factors highlight the need to carefully consider the modelling inference space and are likely to be field or even research question specific.

Most preclinical biomedical research ultimately argues with its relevance for human health. There is substantial evidence that low external validity is an important contributor to failed replication or translation. A plethora of studies in a multitude of models have demonstrated the effects of age, sex, comorbidities, diet, immune status, microbiome, housing conditions, etc., on disease phenotypes or effects of experimental therapies. Studies comparing experimental outcomes and treatments in young, healthy rodents with comorbid, aged or adult immune phenotypes have in the latter shown a reduction of the effect of experimental therapeutics, recapitulating outcomes seen in clinical trials (e.g. hypertension; Macleod *et al*, 2008). However, in many cases, we are lacking prospective evidence for the impact of measures to increase external validity on reproducibility and translation. This is also complicated by the fact that despite improvements, many studies are still lacking internal validity; if results are not internally valid, then assessing external validity is irrelevant. Further, while there are now a multitude of tools to assess internal validity, we are unaware of any validated tools to assess external validity.

## How much external validity is required for translation?

In the light of the plethora of factors challenging generalizability, how much external validity is then required to make translational claims? And how much of this is field or research question specific? For example, do we need to study stroke in one cohort of outbred ageing hypertensive wildling mice that are fed with a high fat diet, while environmental factors are systematically heterogenized across three different laboratories? Clearly no, as countering every known threat to external validity is practically impossible, does not even cover those that are unknown, and goes against the very idea of disease modelling. Animal models provide physical representations of normal and pathological biology that are difficult or impossible to study in humans. They help us dissect and reduce complexity by abstracting and controlling the mechanisms in which we are interested. How then can we resolve the problem of induction? How can we overcome the tension between modelling and the need to generalize the results of this modelling? This should be a pressing question for biomedical researchers concerned about the reproducibility of their work in other laboratories, but particularly for those who make inferences from results obtained in animal experiments to humans, and most importantly, when animal modelling informs the decision to start clinical development (Yarborough *et al*, 2018).

An essential first step is recognizing external validity as a relevant modulator of result reproducibility and translatability, facilitating a more prudent interpretation of the results of animal experiments. In some cases, complementary investigation in animals and the human target population can provide independent evidence underpinning generalization. Biomarkers and imaging can play an important role in this context, as they strengthen inductive reasoning by non‐invasive comparison of equivalence of the behaviour in animal and human (patho)physiological systems.

In scenarios where preclinical studies make therapeutic claims and potentially inform decisions that can lead to interventions in humans, we propose that a minimum set of external validity factors should be considered. In the stroke field, for example, such criteria were proposed by experts from preclinical and clinical academic research as well as the pharmaceutical industry (STAIR). External validity is strengthened when animal model and human target population match with respect to sex and age (equivalent), and results are obtained from at least two independent laboratories and animal facilities in different strains or even rodent species. In Table 1, we propose a set of measures that together can greatly improve external validity and strengthen the evidence base for decision‐making. The results generated by multiple laboratories can be synthesized using preclinical systematic review and meta‐analysis, allowing us to assess external validity across the body of evidence and help inform decisions, such as whether to proceed to clinical development.

We know that these experimental and analysis procedures require substantial resources and pose logistic and potentially regulatory obstacles. Greater awareness of external validity is needed at multiple stakeholder levels to reduce, for example, funding and ethical barriers. More investigation and researcher engagement with methods to optimize experiments, such as factorial designs, can help mitigate necessary resources, particularly the number of animals used, and improve reproducibility. Progressing with therapies based on animal experiments of low external validity to absorption, distribution, metabolism, and excretion (ADME) as well as toxicology studies, is unethical. Not only because of the potential unnecessary suffering or death of experimental animals, but also possible harm to humans when moving into clinical trials.

## Conflict of interest

The authors declare that they have no conflict of interest.
